# Decoding metabolic signatures in Alzheimer’s disease: a mitochondrial perspective

**DOI:** 10.1038/s41420-023-01732-3

**Published:** 2023-12-01

**Authors:** Daniele Bano, Dan Ehninger, Giacinto Bagetta

**Affiliations:** 1 German Center for Neurodegenerative Diseases (DZNE), Bonn, Germany; 2 Department of Pharmacy, Health Science and Nutrition, University of Calabria, Rende, CS Italy

**Keywords:** Alzheimer's disease, Neuroscience

## Abstract

Alzheimer’s disease (AD) is one of the most prevalent age-related neurodegenerative diseases and accounts for the majority of dementia cases worldwide. Tremendous ongoing efforts of basic and clinical research have expanded our knowledge on AD and its complex multifactorial pathogenesis. For sporadic AD, it is widely assumed that silent and early symptomatic stages initiate decades before the irreversible decline in cognitive abilities that ultimately lead to debilitating conditions. In addition to amyloid plaques and tau-containing neurofibrillary tangles as the most prominent hallmarks of AD lesions within the affected brain areas, we now possess a broader collection of pathological signatures that are associated with AD development and progression. In this regard, there is a substantial body of evidence suggesting that hypometabolism occurs in the brains of individuals at the prodromal stage before dementia is diagnosed, which may reflect an early role of metabolic dysfunction in AD. This perspective surveys the vast literature and critically assesses the current evidence demonstrating a mitochondrial contribution to AD. Additionally, we discuss our interpretations of the reported mitochondrial signatures and consider how altered mitochondrial bioenergetics may be an additional risk factor for AD pathogenesis.

## Introduction

Alzheimer’s disease (AD) is an idiopathic, non-communicable neurodegenerative disease with progressive behavioral changes and cognitive impairment, including severe memory decline as the most prominent deficit. As for other neurodegenerative conditions, one of the main features of AD is the long preclinical disease stage in which pathological changes occur in patients, although affected individuals initially do not exhibit striking cognitive symptoms and other clinical manifestations [1, 2]. Compared to other forms of dementia and neurological syndromes, AD is pathologically defined on the basis of neuropathological lesions within affected areas of the brain. Specifically, it is widely reported that neuronal degeneration and death are associated with extracellular amyloid β (Aβ) plaques, intraneuronal tau-containing neurofibrillary tangles and dystrophic neurites highly enriched of hyperphosphorylated tau protein [3–5]. While advancements of new diagnostic approaches have facilitated more robust and accurate classification of AD cases, only a limited number of therapeutic approaches have shown encouraging results in recent trials. Despite some promising developments [6, 7], cognitive improvement of patients remains a matter of intense debate and additional disease-modifying interventions are urgently needed to solve health and social challenges associated with AD [1].

AD is a complex multifactorial neurological disorder of old age, with a prevalence that dramatically increases in people age 65 years or older [1, 8]. As the life expectancy continues to rise worldwide, it is expected that AD will be one of the primary causes of disability and death of elderly people [9], which would lead to serious social and economic consequences for our society [8, 10]. By far, familial early-onset cases of AD (manifesting before 60–65 years) are very rare [11, 12]. These uncommon forms of AD are inherited in an autosomal dominant fashion and are caused by genetic mutations in the presenilin-1, -2 (*PSEN1* and *PSEN2*, respectively) or amyloid-precursor protein (*APP*) genes. Postmortem studies have reported that individuals affected by familial AD feature an abnormal accumulation of Aβ plaques, as well as other neuropathological lesions (e.g., tau-positive neuropil threads and dystrophic neurites) commonly observed in idiopathic AD patients [13–15]. These findings have been pivotal in shaping the amyloid cascade hypothesis, which emphasizes the importance of aberrant Aβ processing as an early upstream event in AD pathogenesis [16, 17]. Moreover, genome-wide association studies have pinpointed several genetic risk factors for sporadic AD, consistently highlighting the robust association between AD and certain variants of apolipoprotein E (APOE) and triggering receptor expressed on myeloid cells 2 (TREM2) among others [18–21].

Another characteristic feature of AD is the progressive decline of glucose metabolic rate in certain areas of the brain. In patients with early signs of mild cognitive impairment, multiple longitudinal studies indicate that brain hypometabolism may correlate with the development of tau deposition and atrophy of the temporal and parietal lobes [22–24]. Despite the adult brain accounts for ~2% of the total body mass, it consumes a large portion of glucose and other carbon substrates that circulate in the blood and are metabolized via glycolysis and mitochondrial oxidative phosphorylation (OXPHOS) at different rates by neurons, glia and other resident cells within the central nervous system [25–27]. Based on these considerations and experimental data in model organisms [28, 29], there is compelling evidence that aberrant metabolism and defective mitochondrial bioenergetics may be relevant for AD onset and progression.

With this perspective, we aim to assess the most convincing findings suggesting a mitochondrial involvement in AD pathogenesis.

### Mitochondria and AD: shareholders or mere bystanders?

Mitochondria are intracellular double membrane organelles providing energy in the form of ATP as well as a variety of metabolic intermediates that are exported to the cytosol or transferred to other intracellular organelles [30]. The conversion of carbon substrates into ATP relies on a series of evolutionarily conserved redox reactions, in which electrons are transferred from reduced donor molecules (e.g., from glucose to NADH) to oxygen as terminal acceptor. In the vast majority of eukaryotic cells [31], the electron transport chain (ETC) comprises four respiratory complexes and two electron carriers that sustain the proton pumping across the inner mitochondrial membrane. By building up the mitochondrial membrane potential, the ETC promotes the activity of the ATP synthase (Complex V) and therefore the OXPHOS pathway that synthesizes ATP from ADP and inorganic phosphate. Additionally, mitochondria host several enzymes involved in the conversion of complex molecules into metabolic precursors (e.g., intermediates of the citric acid cycle or amine from the urea cycle) [30, 32–34]. As highly dynamic organelles, mitochondria can regulate ion homeostasis (e.g., calcium), cell death and intracellular signaling (e.g., reactive oxygen species, calcium, cAMP) by modulating their metabolic behaviors and molecular features [33, 35–37].

Consistent with these pleiotropic functions, aberrant mitochondrial activities have been linked to several human disorders, including metabolic syndromes and neurodegenerative diseases. However, while the genetic link between mitochondria, inherited neuropathies and metabolic disorders is well-described and widely observed in clinical practice and experimental biology [38–42], a conclusive causal connection between mitochondria and AD is less well defined, especially if compared to other neurodegenerative diseases, such as Parkinson’s disease (PD) and amyotrophic lateral sclerosis (ALS) [43–47]. Recent clinical findings have reported that missense mutations in the gene encoding pitrilysin metallopeptidase 1 (PITRM1, also known as presequence protease) may cause the accumulation of Aβ-positive deposits [48–50]. A study of a single Norwegian family revealed that patients carrying pathogenic *PITRM1* mutations develop progressive spinocerebellar ataxia and functional changes of mitochondrial bioenergetics in muscle biopsy. In transgenic mice, yeast and cultured cells, PITRM1 deficiency seems to negatively affect the ability of cells to degrade Aβ peptide [48, 51]. As PITRM1 is a mitochondrial matrix enzyme involved in the cleavage of targeting sequences after protein translocation, one hypothesis is that PITRM1 can directly participate in the digestion and clearance of Aβ species that are eventually imported in mitochondria [48, 52]. Although the contribution of mitochondria in Aβ degradation remains a matter of intense investigation and debate [53–56], in-depth clinical assessments of patients carrying pathogenic *PITRM1* mutations confirmed low levels of Aβ_1–42_ in the cerebrospinal fluid (CSF) that are comparable to those in AD patients [48]. Future studies will determine whether PITRM1-dependent Aβ lesions are detectable in the human brain and form deposits in distinct regions of the nervous system or spread uniformly in all brain area.

Over the past years, many genome-wide association studies (GWAS) have reported single nucleotide variants associated with a higher risk of late-onset AD. Some of these genetic variants are located near genes contributing to mitochondrial bioenergetics, such as *ECHDC3*, *COX7C* and *NDUFAF6* genes encoding enoyl-CoA hydratase domain-containing protein 3, cytochrome c oxidase subunit 7C and NADH:ubiquinone oxidoreductase complex assembly factor 6, respectively [20, 57, 58]. Despite the lack of compelling causative genetic evidence for a link between mitochondria and AD, an increasing number of studies have reported changes in brain glucose and oxygen metabolism as well as mitochondrial respiratory defects and/or morphological abnormalities in tissues exhibiting typical AD-related neuropathological changes [26, 28, 44].

Using positron emission tomography (PET), longitudinal assessments of patients with early AD have recently revealed progressive reduction of Complex I radioligand binding [59]. These data may suggest that aberrant OXPHOS system correlates with early signs of cognitive decline in individuals with AD. While these findings may depict mitochondrial impairment as a consequence, rather than a cause of AD, other postmortem tissue assessments support alternative views. In this regard, gene set enrichment analyses suggest that AD patients exhibit aberrant expression of mitochondrial OXPHOS subunits or molecular factors contributing to mitochondrial proteostasis [54]. Consistently, single-nuclei sequencing of human entorhinal cortex and subsequent gene set enrichment analysis show a tendency toward downregulation of genes encoding mitochondrial respiratory complex subunits in certain subpopulations of neurons of AD patients [60]. Similar transcriptional signatures are also observed when transcriptomes of posterior cingulate astrocytes were performed using brain tissues from AD patients and age-matched healthy subjects [61]. These data suggest that not only neurons, but also glia may experience metabolic stress as a consequence of mitochondrial OXPHOS defects associated with AD-related processes.

Using isobaric labeling for quantitative proteomics, it was shown that significant changes in Complex I subunits as well as a few additional components of other respiratory complexes can be detected in postmortem brain tissues (medial frontal gyrus) of AD patients compared to aged-matched non-demented women [62]. As these defects in Complex I were observed in individuals aged 68 years, it is nevertheless difficult to conclude if these changes were due to the loss of long-lived postmitotic neurons or to compensatory processes that are linked to age-related hypometabolism or other pathogenic events (e.g., neuroinflammation) associated with AD. Consistent with the idea that mitochondria defects may be considered early signatures and possibly used as biomarkers of AD [63], widespread alterations of the mitochondrial proteome have been detected by independent proteomic analyses. These changes seem to be significant in the brain cortex, cerebrospinal fluid and serum of patients with mild cognitive impairment (MCI). Conversely, a very recent proteomic comparison has shown that substantial changes of the mitochondrial proteome are detectable only in brain tissues of advanced AD patients, while only a few mitochondria proteins were upregulated in early-stage AD [64].

Taken together, these experimental data indicate a strong association between aberrant mitochondrial bioenergetics and AD, although its precise contribution to the development of the disease requires further mechanistic evidence.

## Conclusions and perspectives

**Fig. 1:**
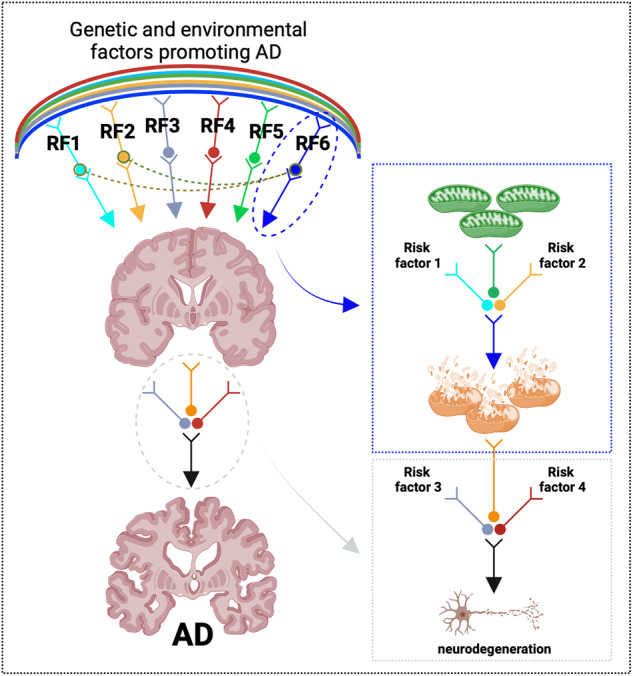
AD results from a complex interplay of various factors associated with aging. As such, a range of genetic, epigenetic and environmental factors (identified as RF1 to RF6 in the schematic) may promote neurodegenerative processes that ultimately cause cognitive impairment. Alterations of mitochondria have been detected in brains of AD patients. These mitochondrial changes may depend on various upstream and/or modifying processes (depicted as risk factor 1 and 2), which could cumulatively compromise mitochondrial function. While severe mitochondrial lesions may be sufficient to trigger a neurodegenerative cascade, it is also possible that additional factors (indicated here as risk factor 3 and 4) are required to irreversibly undermine neuronal survival. Created with Biorender.com.

This perspective aims to survey the currently available data obtained from recent studies of subjects at risk of AD as well as postmortem assessments of tissues from healthy and cognitively impaired donors. We recognize the impressive advances in the field, despite the substantial limitations in current detection methods and the availability of properly collected postmortem samples. We cover studies of the last decade and report the most convincing data suggesting a correlation between the expression changes of mitochondrial components and the progressive age-related development of AD. On purpose, we did not review the abundant literature on transgenic mice and other in vivo and in vitro experimental models, from which we learnt how modulation of mitochondrial behaviors can influence several aspects of Aβ and tau pathology. Despite the uncertainty about the contribution of mitochondria in AD, we think that altered glucose and oxygen metabolism may develop first and become a prominent signature during the course of aging. Due to circulatory and/or other risk factors, the diminished supply of substrates would then undermine mitochondrial bioenergetics and lead to energy production defects, ROS generation, aberrant intracellular signaling and impaired mitochondrial biosynthetic activities. In such a pathological scenario, defective mitochondria would compromise even further the resilience mechanisms of neurons and neural circuitry that, in aging, could become more susceptible and vulnerable to injuries [27, 65–67]. Rather than an upstream event in AD pathogenesis, aberrant mitochondrial bioenergetics may occur as a result of age-related metabolic dysfunction, with consequent defects in mitochondrial outputs that would reinforce a vicious loop undermining neuronal survival (Fig. 1). We look forward to future studies that are able to conclusively define the temporal participation of mitochondria to AD pathogenesis as well as the conditions by which modulation of mitochondrial bioenergetics may be of benefit in AD.
